# Cytological and Transcriptional Dynamics Analysis of Host Plant Revealed Stage-Specific Biological Processes Related to Compatible Rice-*Ustilaginoidea virens* Interaction

**DOI:** 10.1371/journal.pone.0091391

**Published:** 2014-03-19

**Authors:** Jinquan Chao, Jie Jin, Dong Wang, Ran Han, Renshan Zhu, Yingguo Zhu, Shaoqing Li

**Affiliations:** 1 State Key Laboratory for Hybrid Rice, College of Life Science, Wuhan University, Wuhan, China; 2 Department of Statistics, University of Nebraska, Lincoln, Nebraska, United States of America; Wuhan University, China

## Abstract

Rice false smut, a fungal disease caused by *Ustilaginoidea virens* is becoming a severe detriment to rice production worldwide. However, little is known about the molecular response of rice to attacks by the smut pathogen. In this article, we define the initial infection process as having three stages: initial colonization on the pistil (stage 1, S1), amplification on the anther (stage 2, S2) and sporulation in the anther chambers (stage 3, S3). Based on the transcriptome of rice hosts in response to *U. virens* in two separate years, we identified 126, 204, and 580 specific regulated genes in their respective stages S1, S2, and S3, respectively, by excluding common expression patterns in other openly biotic/abiotic databases using bioinformatics. As the disease progresses, several stage-specific biological processes (BP) terms were distinctively enriched: “Phosphorylation” in stage S1, “PCD” in S2, and “Cell wall biogenesis” in S3, implying a concise signal cascade indicative of the tactics that smut pathogens use to control host rice cells during infection. 113 regulated genes were coexpressed among the three stages. They shared highly conserved promoter cis-element in the promoters in response to the regulation of WRKY and Myb for up-regulation, and ABA and Ca^2+^ for down regulation, indicating their potentially critical roles in signal transduction during rice-*U. virens* interaction. We further analyzed seven highly regulated unique genes; four were specific to pollen development, implying that pollen-related genes play critical roles in the establishment of rice susceptibility to *U. virens*. To my knowledge, this is the first report about probing of molecular response of rice to smut pathogen infection, which will greatly expand our understanding of the molecular events surrounding infection by rice false smut.

## Introduction

*Ustilaginoidea virens* is a fungal pathogen that causes rice false smut, an epidemic disease responsible for severe yield losses of rice crops around the world [1]–[3]. Rice severely infected by rice false smut present a dark green spore ball in spikelets after heading. The mature green balls eventually break, resulting in the release of mist-like yellow spores. As a consequence, released spores become a source of secondary infection in rice fields, and the contaminant ustilotoxins they produce are hazardous to mammals [4]. In China, it has been estimated that one third of rice cultivation areas are damaged by rice false smut [2]. In some regions, rice false smut disease has become as severe as that of blast and sheath blight [5],[6]. Rice false smut represents a new threat to rice production around the world because all current commercial rice varieties are susceptible. Few resistant germplasts are available in breeding programs, although it was claimed that the rice varieties IR28 and Lemont carry resistant QTLs against rice false smut [2]. However, we found that the F_1_ progeny derived from these resistant strains are still susceptible to rice false smut (data not shown), suggesting that these QTLs cannot be used as resources for smut-resistant breeding programs.

The outbreak of this disease has necessitated a greater understanding of its diversity, infection process, heredity, toxicity and overall damage to rice production [1]. Previous studies have suggested that the primary infection sites for the pathogen are the upper parts of the stamen filaments located between the ovary and the lodicules. Additionally, the floral organ is later enmeshed by hyphae [7]. However, infection occurs only at the booting stage, which strongly hinders precise probing for details of the infection and regulatory networks involved in the rice response to *U. virens* invasion. With current techniques, it is very difficult to isolate infected spikelets, even on the inoculated panicles. The disease can only be identified when a visible yellowish spore ball protrudes from the glume after heading, which facilitates observations of infection progression in later stages [7]. As a result of these characteristics, the molecular details of the early infection process have remained elusive. The lack of known resistance genes and information about molecular host responses to *U. virens* attack greatly hinders the development of strategies to create resistant germplasm for rice false smut disease. Due to the significance of rice production, understanding the molecular mechanisms underlying infection by smut pathogen is of utmost importance. In general, the outcome of plant-pathogen interactions depends on a molecular interaction between the two organisms, with the pathogen attempting to control the plant cell to establish an environment conducive to its replication [8].

A transcriptome represents a comprehensive set of transcribed loci throughout the genome, which provides important insights into the functional elements, the expression patterns, and the regulation of transcribed regions of the genome under different conditions. Zhang *et al.* [9] recently elucidated pathogen specific molecular strategies by monitoring the dynamic expression profiles of both *Fusarium graminearum* and infected wheat coleoptiles using microarrays. Digital gene expression tag profiling (DGE) based on RNA-Seq presents huge potential for exploring biological questions because of its the high levels of reproducibility for both technical and biological replicates and of its capability to produce large amounts of sequence data for analysis [10],[11]. Comparing significantly different expressional profiles in different tissues allows us to probe the target expression loci, as well as elements related to a specific stimulus or environment. This powerful approach has been successfully employed in the analysis of plant disease defense systems [12]–[14].

In the current study, we investigated the early progression of smut disease following inoculation of plants with spores and analyzed expression profiles of spikelets infected with *U. virens* using DGE. Stage-specific biological processes related to compatible plant-pathogen interactions were identified which represent novel elements involved in communication between rice and the *U. virens* pathogen. These findings will shed light on our understanding of the molecular events surrounding infection by rice false smut.

## Materials and Methods

Ethics statement: No specific permissions were required for sample collection at the locations mentioned in this manuscript. The fungus used in this study is not an endangered or protected species.

### Plant materials

The false smut-susceptible rice cultivar 93-11 (*Oryza sativa* L. ssp. *indica*) was used in this study. Rice plants were planted in a greenhouse under a 14-h-light/10-h-dark regime in the summer season of 2010–2011. After inoculation, rice plants were kept at a constant 20/28 °C night/day cycle at 85% relative humidity.

### Inoculation of rice smut pathogen and spikelet sampling

The fungal isolate *Guangxi-2* was purified from the spore-ball of rice false smut collected from Guangxi province, China. Briefly, sterilized smut balls were inoculated on PSA medium plates at 28°C until the appearance of mycelium. Then, *U. virens* was purified by single spore and transferred individually to flasks containing PS medium for growth in a rotary incubator at 200 rpm, 28 °C for 6–8 days. The culture was filtered using 4–6 layers of cheesecloth, conidia were collected conidia by centrifugation, and then spores were diluted with ddH_2_O to one million per milliliter.

Inoculation followed the methods of Li *et al.* [2]. 2 ml of inoculum suspension were injected into swollen sheaths of flag leaves on the main stems of rice one week before heading, and water was used for control plants. After heading, the treated spikelets were carefully observed; diseased spikelets with identical inflorescence differentiation stages were collected and sorted into three groups following infection progression for cytological and molecular analysis [15].

### RNA extraction, library construction and sequencing

Total RNA was extracted from frozen samples using Trizol (Promega, Germany) according to the manufacturer's protocol. Library construction and sequencing were performed at Beijing Genomics Institute (Shenzhen, China). Briefly, 2 μg total RNA of each group was used for mRNA capture with magnetic oligo-dT beads. Addition of fragmentation buffer separated mRNA into short fragments (about 200 bp). First strand cDNA was synthesized by random hexamer-primer using the mRNA fragments as templates. Buffer, dNTPs, RNase H and DNA polymerase I were added to synthesize the second strand. Double stranded cDNA was purified with a QiaQuick PCR extraction kit and washed with EB buffer for end repair and single nucleotide A (adenine) addition. Finally, sequencing adaptors were ligated to the fragments. The required fragments were purified by agarose gel electrophoresis and enriched by PCR amplification. The library products were pair-end sequenced (length 75 bp) via Illumina HiSeq 2000 (Table S1 in File S1).

### Analysis of DGE tag and categorization of differential gene expression

The original image datasets were transferred into sequence datasets by base calling. Prior to mapping reads to the reference database, we filtered all sequences to remove adaptor sequences, low quality sequences (tags with unknown sequences ‘N’), empty tags (sequence with only adaptor sequences but no tags), low complexity sequences, and tags with only one copy (potential sequencing errors). A reference database was generated by integrating the indica rice genome (The Beijing Gene Finder, http://bgf.genomics.org.cn/) into japonica rice full-length cDNAs from the KOME (Knowledge-based *Oryza* Molecular Biological Encyclopedia) site (http://cdna01.dna.affrc.go.jp/cDNA/) with a 95% threshold value as described [16] and clean reads were mapped to the reference database using SOAPaligner/soap2, allowing for a two base pair mismatch for elimination reads from the pathogen.

To compare DGE genes expression across samples (CK vs S1, CK vs S2, CK vs S3), raw clean tags in each library were normalized to Tags Per Million (TPM) to obtain normalized gene expression levels. DGE genes were deemed with a FDR value ≤0.001 and |log_2_ Ratio|≥1 in sequence counts across libraries. All DGE genes were categorized by MapMan software.

### Semi-quantitative RT-PCR, quantitative PCR (qPCR) analysis

To verify the DGE results, qPCR was carried out on the RNA samples yielded from the DGE experiments on a Lightcycler480 (Roche) using the SYBR PrimeScript RT-PCR Kit (TaKaRa Biotechnology, China). The average threshold cycle (CT) was calculated for each cDNA sample. The initial template amount was calculated by relating CT values to a standard curve constructed by amplifying genomic DNA of known concentrations. Expression values were normalized for differences in cDNA input using parallel reactions employing primers designed against a reference gene, *OsUBI*, whose expression was not altered by *U. virens* infection.

For the semi-quantitative RT-PCR reactions, the numbers of reaction cycles was 25–33. *OsUBI* was used to normalize cDNA loading. The amplified products were resolved by 1.5% (w/v) agarose gel electrophoresis and visualized with ethidium bromide using AlphaEase software (Alpha Innotech, CA). PCR primers were refered to Table S2 in File S1.

### Publically available rice bio-/abiotic microarrays and global analysis gene expression patterns on the web

For studying rice abiotic stresses, the database (cold, heat, salt and drought) “Common and distinct organ and stress responsive transcriptomic patterns in *Oryza sativa* and *Arabidopsis thaliana*” (http://www.biomedcentral.com/1471-2229/10/262) was used. For studying biotic stresses, the raw data for Affymetrix GeneChips as GSE29967 (brown planthopper), GSE11025 (rice stripe virus), GSE28012 (rice stripped stem borer), GSE7256 (rice blast) were downloaded from the Gene Expression Omnibus (http://www.ncbi.nlm.nih.gov/geo/). For each experiment, microarray data were normalized using the RMA method [17]. All numerical calculations were carried out with the R statistical software package (http://www.r-project.org). Two web sites (http://crep.ncpgr.cn/crep-cgi/query_by_tree.cgi and http://www.ricearray.org/expression/expression.php) integrated the vast amount of rice microarray data that was used for analysis of global gene expression patterns.

### Scanning electron microscopy

The infection process of rice false smut was observed using scanning electron microscopy (SEM). Briefly, freshly sampled diseased spikelets were fixed with FAA, then dehydrated through a ethanol gradient (50, 70, 80, 90 and 100%), dried by a critical-point drying method with liquid CO_2_ coated with gold, and examined with a scanning electron microscope (JSM-5410LV; JEOL Ltd., Japan) at an accelerating voltage of 20 kV.

### Stress treatment with biotic/abiotic factors

Four-leaf rice seedlings were used for *U. virens*-reactive gene expression analysis under biotic and abiotic stresses. Treatment conditions of salt, cold, drought and submergence followed the method of Qi *et al.* [18], heat stress followed the method of Jin *et al.* [19], and inoculation of *Magnaporthe oryzae* and *Xanthomonas oryzae* pv. *Oryza* (*Xoo*) followed the method of Qiu *et al.* [20].

### Starch and soluble sugar content determination and statistics

Starch measurement followed the method of Rufty and Huber [21]. Soluble sugar analysis was conducted according to the method of Ebell [22].

All assays on plant biological and biochemical characteristics were performed at least three times, and values reported are the mean of three replicates ±SD.

## Results

### Distinctive infection stages of *U. virens* in rice spikelets

**Figure 1:**
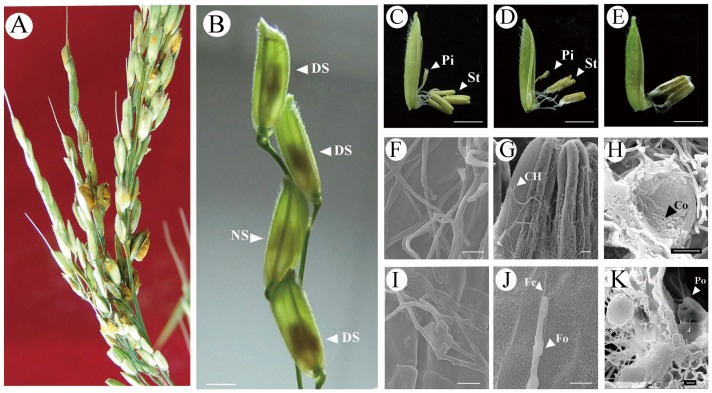
Pathogenic development of rice false smut. (**A**) Phenotypes of rice false smut disease. Arrows in Fig. A indicate a mature false smut ball. (**B**) Early development of rice false smut disease. DS, means the diseased spikelets, and NS, means normal spikelets. (**C–K**) Cytological observation and electronic scanning of floral organs infected with *U. virens*. (**C**) Observation of the infected floral organs at stage S1. (**D**) Observation of the infected floral organs at stage S2. (**E**) Observation of the infected floral organs at stage S3. (**F, I**) SEM observation hyphae on style (stage 1). (**G, J**) SEM observation hyphae on stamen (stage 2). (**H, K**) SEM observation the innate of infected stamen at stage 3. Pi, indicates pistil; St, indicates stamen; Fo, represents “foot-like”structure; Fe, represents “feelers-like” structure. Co, represent “conidia”; Po, represent “pollen”. Bar = 1 cm in Fig. B, C, D, E; Bar = 100 μm in Fig. G, J; Bar = 10 μm in Fig. F, H, I, K.

*U. virens* infects only the reproductive tissues, and the diseased spikelets can be found scattered along the inoculated panicles after heading (Fig. 1A and B). Cytological observation of the infection progression revealed that *U. virens* infection was a stepwise invasion process, which can be divided into three distinctive stages based on the morphology of the infected floral organs (Fig. 1C–E). First, stage S1 is characteristic of the initial colonization hyphae. At stage S1, hyphae were observed living on the pistils in the infected spikelets, and the two pistils were gradually intertwined by the fungal hyphae (Fig. S1A in File S1). The pathogenic special organ infection cushion was formed on the column (Fig. 1I). At stage S2, a mycelial mass proliferated at the bottom of the ovary and filament, and climbed up to the anther using specialized “feet” and “feelers” (Fig. 1G, J). Finally, at stage S3, the floral organ was covered with a dense layer of fungal hyphae (Fig. S1B in File S1) which later entered into the anther chamber by piercing through the anther wall at the weak connectivum (Fig. S1C in File S1). Masses of conidia were found wrapped on the surfaces of pollen grains, some of which were collapsed (Fig. 1H, K). These findings are inconsistent with previous references that suggest that the initial colonization of the rice smut pathogen occurs on the anther styles [7]. We believe that these differences may be due to the fact that previous observations were recorded at later stages of disease progression.

### Transcriptional profiles were extensively modified during infection by *U. virens*

To gain further insight into the interactions between the host plant and *U. virens*, we evaluated rice transcriptional profiles during *U. virens* infection. RNA was isolated from tissues collected from the control and infected spikelets at three stages for DGE analysis using an upgraded Solexa/Illumina's DGE system. To minimize the effects resulting from sampling and the environment, samples were collected from different years (2010∼2011) and were analyzed independently (Fig. S2 in File S1, Table S1 in File S1). In 2010, 34771 transcripts were detected in the control samples, while 34201, 33458, and 32599 transcripts were detected from samples collected at stages S1, S2, and S3, respectively (NCBI accession numbers: GSE32010). In 2011, the numbers of transcripts detected were 33020 in the control samples, and 32610, 31818, and 30994 in diseased samples taken at stages S1, S2, and S3, respectively (NCBI accession numbers: GSE39049). Under the limit rule with FDR≤0.001 and |log_2_ Ratio|≥1, 1251 (S1), 2161 (S2) and 3734 (S3) differentially expressed genes were detected in 2010, and in 2011 2342 (S1), 2121 (S2), and 3090 (S3) differentially expressed genes were detected (Fig. S3 in File S1).

**Figure 2:**
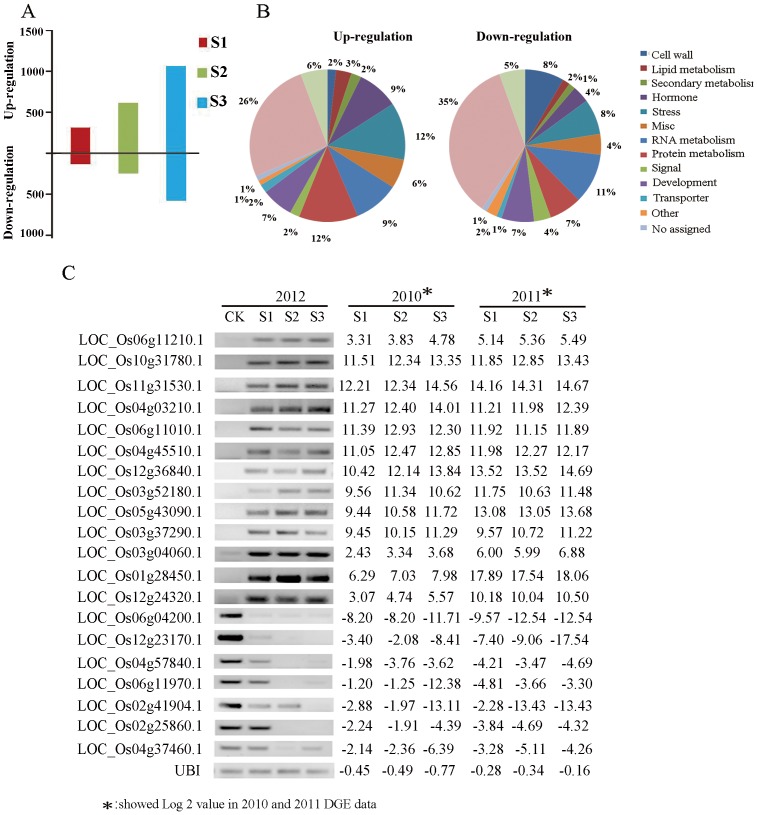
Integrative analysis of differentially expressed genes across both years. (**A**) Differentially expressed genes monitored across both years. Numbers on the Y-axis represent the regulated gene numbers. (**B**) Functional categorization of the all differentially expressed genes across both years using MapMan software. (**C**) Verification of the special genes using RT-PCR amplification of the inoculated samples in 2012. PCR amplifications were carried out with 30 cycles, * showed Log_2_ values in 2010 and 2011 DGE data.

To reduce background noise from fluctuating gene expression in plants under different growth environments, only genes showing similar expression patterns at each stage in both years were chosen for further analysis. Based on this criterion, 423, 879 and 1620 differentially expressed genes were detected across both years at stages S1, S2 and S3, respectively (Fig. 2A). To further verify these data, twenty randomly selected genes were amplified by RT-PCR with the *U. virens* infected spikelets collected in 2012; they all showed similar expression patterns to samples analyzed in 2010 and 2011 (Fig. 2C).

Among the differentially expressed genes identified, most were up-regulated by pathogen infection. As the stages of the disease progressed, the ratio of up-regulated genes to the whole set of modified genes decreased from 69.8% at S1 to 65.8% at S3. The differentially expressed genes were divided into different functional categories using the “Overview” function of MapMan software. Categories such as “Protein metabolism”, “RNA metabolism” and “Stress” take a dominant position in the induced transcripts resulting from infection with *U. virens* (Fig. 2B). These results indicate that genes modulated by the rice smut pathogen are mainly involved in plant defense responses and the regulation of gene expression.

### Specific gene expression related to the infection of *U. virens*

Substantial changes in gene expression associated with various biotic stresses are usually induced by the host plant to activate any and all defenses available to respond to a pathogen invasion [14],[23]. However, only a few of the special differentially expressed genes comprise the core web that mediates initial interactions between a host plant and a pathogen. It is hard to determine the identity of these unique genes to a pathogen just by comparing a control and a disease treatment as there are many genes modulated in response to stress that are not specific to the pathogen response. However, by using public biotic and abiotic stress gene expression databases it is possible to eliminate many of the general stressors. Thus, we compared our data to that of openly available biotic and abiotic databases, and considered the genes that were found to be significantly associated with the stress response in previous studies (P≤0.05, |log_2_ Ratio|≥1) as general stress-related genes. The remaining genes were classified as *U. virens* special response genes. The general stress related genes represented 70.2%, 65.2% and 64.0% of the whole set of induced genes at stages S1, S2 and S3 respectively, and about 80% of them fell in the up-regulation gene set during *U. virens* infection (Table 1), implying that most of the over expressed genes are in fact generally involved in the stress response.

**Table 1:** Analysis of the regulated genes in rice during infection of *U. virens*

Categories of regulated genes	Stage S1			Stage S2			Stage S3		
	Total	Up	Down	Total	Up	Down	Total	Up	Down
The whole regulated gene number	423	300	123	873	602	271	1611	1060	551
Relative ratio (%)		69.8%	30.2%		69.0%	31%		65.8%	34.2%
Specially regulated gene number	126	58	68	304	136	168	580	243	337
Relative ratio (%)		46.0%	54.0%		44.7%	55.3%		41.9%	58.1%
Generally regulated gene number	297	242	55	569	466	103	1031	817	214
Relative ratio (%)		81.5%	18.5%		81.9%	18.1%		79.2%	20.8%

Note: up, means up-regulation; down, means down-regulation.

**Figure 3:**
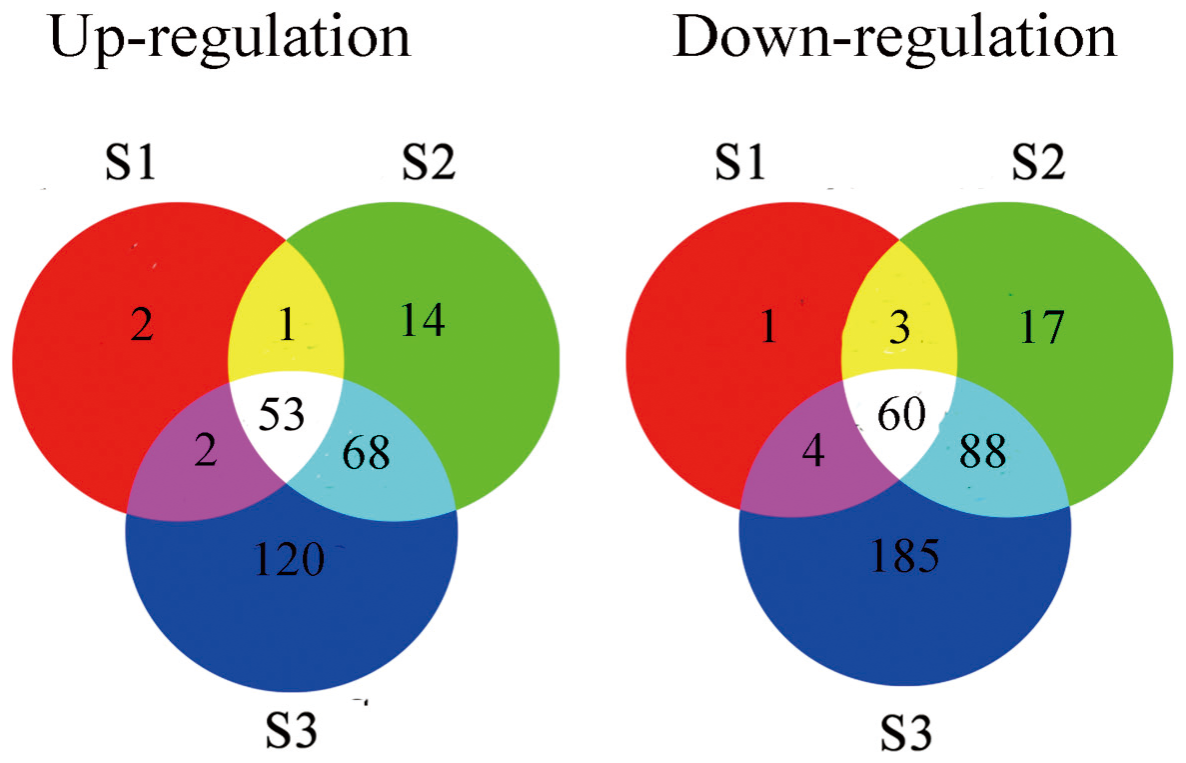
Comparison analysis of the special DE genes for rice among three stages.

After excluding the general stress regulation genes from whole transcription profiles, 126, 304, and 580 genes were specifically co-related to *U. virens* invasion at stages S1, S2, and S3, respectively (Fig. 3). They represented about only one third of the whole set of expression changes in the three stages (Table 1). In contrast to the general stress expression set, the up-regulation set of disease specific gene expression levels accounted for 46%, 44.7% and 41.9% of the *U. virens* specifically induced transcripts in stage S1, S2 and S3 respectively (Table 1).

### Pathogen specific genes show a distinctive stage enrichment profile

To better understand the stage-specific expression profiles of rice after *U. virens* infection, we analyzed the specifically-regulated genes for BP terms using AgriGO singular enrichment analysis. The results showed that a total of 33 up-regulated BP terms and 19 down-regulated BP terms were detected in the three stages (Table 2). As the disease developed, the number of enriched BP terms gradually increased from 15 at stage S1 to 26 at stage S2, and 47 at stage S3. Among those up-regulated, there were a total of 33 GO biological processes enriched in at least one of the three disease stages. Of these, 13 out of 14 enriched BP terms in S1 were overlapped by those found in stages S2 and S3. Similarly, all 19 enriched BP terms in S2 were completely comprised by that of enrichment in S3, reflecting a continuous activation of defense systems to cope with pathogen attack during smut pathogen disease progression.

**Table 2:** Enriched stage-specific BP terms regulated by *U. virens* infection (FDR<0.05)

GO number	Annotation	FDR-value		
		S1	S2	S3
**Up-regulation**				
GO:0006508	proteolysis	0.025		
GO:0006468	protein amino acid phosphorylation	5.40E-05	2.20E-08	1.90E-21
GO:0016310	phosphorylation	5.40E-05	5.50E-08	3.20E-20
GO:0043687	post-translational protein modification	5.40E-05	1.00E-07	2.60E-19
GO:0006796	phosphate metabolic process	5.40E-05	1.00E-07	3.60E-19
GO:0006793	phosphorus metabolic process	5.40E-05	1.00E-07	3.60E-19
GO:0006464	protein modification process	5.40E-05	1.30E-07	3.50E-18
GO:0043412	macromolecule modification	5.40E-05	1.80E-07	8.40E-18
GO:0044267	cellular protein metabolic process	0.0016	1.20E-05	2.50E-14
GO:0019538	protein metabolic process	6.30E-05	2.40E-05	3.80E-13
GO:0043170	macromolecule metabolic process	0.0016	0.0019	3.30E-07
GO:0044260	cellular macromolecule metabolic process	0.038	0.0037	1.30E-06
GO:0044238	primary metabolic process	0.0078	0.002	1.30E-06
GO:0008152	metabolic process	0.0051	0.00045	3.20E-06
GO:0012501	programmed cell death		0.00021	1.40E-07
GO:0006915	apoptosis		0.00021	1.40E-07
GO:0008219	cell death		0.00021	1.70E-07
GO:0016265	death		0.00021	1.70E-07
GO:0009987	cellular process		0.0024	4.50E-06
GO:0006952	defense response		0.00062	4.00E-05
GO:0009856	pollination			4.50E-06
GO:0008037	cell recognition			4.50E-06
GO:0009875	pollen-pistil interaction			4.50E-06
GO:0048544	recognition of pollen			4.50E-06
GO:0022414	reproductive process			5.30E-06
GO:0051704	multi-organism process			9.00E-06
GO:0000003	reproduction			1.70E-05
GO:0044237	cellular metabolic process			8.50E-05
GO:0032501	multicellular organismal process			9.10E-05
GO:0007154	cell communication			0.0003
GO:0006457	protein folding			0.0032
GO:0006950	response to stress			0.0062
GO:0050896	response to stimulus			0.011
**Down-regulation**				
GO:0005975	carbohydrate metabolic process	0.047	0.018	0.00065
GO:0006644	phospholipid metabolic process		0.0023	0.0019
GO:0019637	organophosphate metabolic process		0.0023	0.0023
GO:0015672	monovalent inorganic cation transport		0.018	
GO:0044255	cellular lipid metabolic process		0.018	
GO:0016051	carbohydrate biosynthetic process		0.018	
GO:0030001	metal ion transport		0.044	
GO:0042545	cell wall modification			0.00018
GO:0006468	protein amino acid phosphorylation			0.00065
GO:0071555	cell wall organization			0.00087
GO:0043687	post-translational protein modification			0.00087
GO:0006650	glycerophospholipid metabolic process			0.001
GO:0046486	glycerolipid metabolic process			0.001
GO:0016310	phosphorylation			0.0011
GO:0006464	protein modification process			0.0017
GO:0043412	macromolecule modification			0.0022
GO:0006796	phosphate metabolic process			0.0023
GO:0006793	phosphorus metabolic process			0.0023
GO:0071554	cell wall organization or biogenesis			0.0064

Note: FDR, False discovery rate.

**Figure 4:**
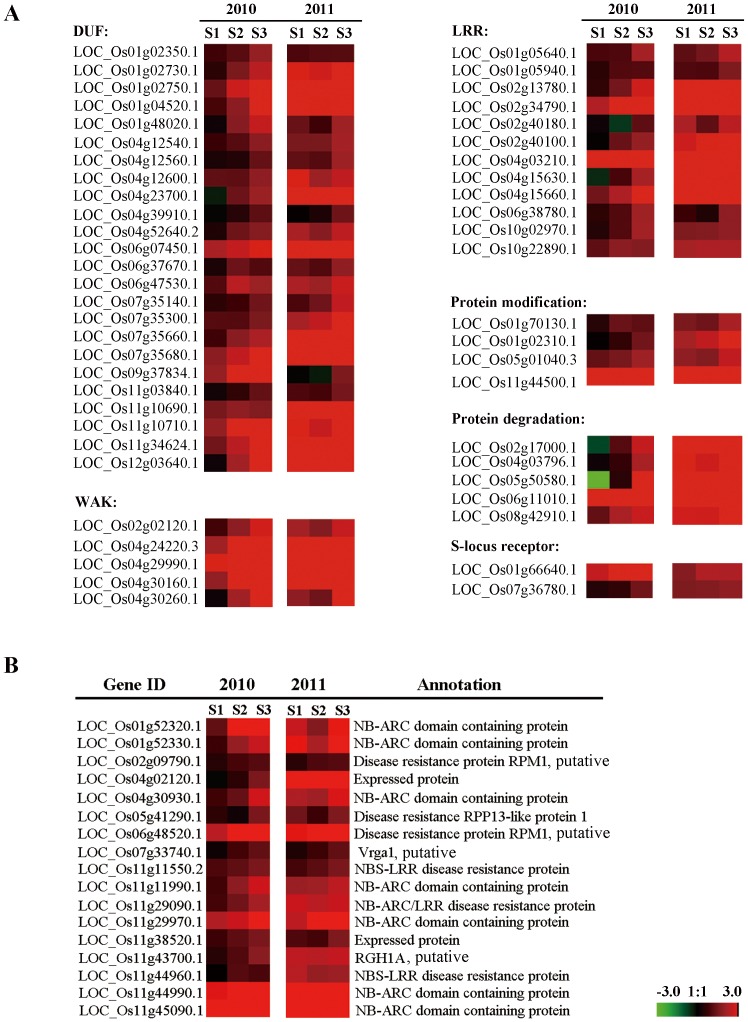
Heat map of the stage-specific genes involved in BP terms of “protein modification” and “cell death”. (**A**) Categories and heat map of “protein modification” genes. (**B**) Categories and heat map of “cell death” genes. Colours bar represent expression levels of each gene which are either up-regulated (red) or down-regulated (blue).

In S1, 13 out of 14 BP terms were up-regulated. Among these, apart from “Proteolysis”, “Primary metabolic process” and “Metabolic process” BP terms, the other four BP terms were related to the phosphate metabolic process, and seven BP terms were related to protein modification. They were conserved among three stages, and could be categorized into WAK, LRR and DUF etc. subfamilies according to structures (Fig. 4A). This suggests that protein modification and phosphorylation related processes play critical roles in the mediation of defense against the rice false smut pathogen by host cells at the beginning of infection.

As the disease progresses to S2, six more novel BP terms related to cell death were enriched. These are composed of 17 genes including *RPM1*, *RPP13*, *RGH1A*, and *vrga1* homology which have been characterized as plant disease resistance genes, as well as 10 members of the NBS-LRR/NB-ARC family (Fig. 4B) [24],[25],[26],[27]. In parallel, the enriched BP terms in the down-regulated set rose to seven, with four of these being specific to S2, including “Metal ion transport”, “Monovalent inorganic cation transport”, “Cellular lipid metabolic process” and “Carbohydrate biosynthetic process”. This implies that antheric *U. virens* infection activates host cell death programs in an attempt to thwart pathogen colonization. It is well known that hypersensitive responses are activated at the infection site early on, which gives rise to PCD, ROS burst, and a concurrent disturbance of metal ion flow and carbohydrate biosynthesis [8],[28].

In S3, 32 different BP terms were enriched among the up-regulated set, of which 13 terms including “Cell recognition”, “Response to stress”, “Pollination” etc. were unique to this stage. These data reveal the molecular responses of pollen to invasion by the smut pathogen, and they are consistent with cytological observations that the pathogen hyphae intertwine with microspores and large amounts of pathogenic conidia are produced in the anther chambers. Of the down-regulated set of genes, 12 out of 15 enriched BP terms were determined to be specific to this stage. These BP terms were mainly involved in processes related to cell wall biogenesis and glycerol metabolism, including “Cell wall modification”, “Cell wall organization or biogenesis”, and “Glycerophospholipid metabolic process” etc., reflecting the fact *U. virens* infection inhibits cell wall biogenesis and alters carbohydrate metabolism in the anthers. This is consistent with cytological observations of pollen collapse and huge amount of conidia in anther chambers.

### Conserved *cis*-acting elements shared by the commonly regulated genes among three stages

Among all genes that were differentially expressed during *U. virens* infection, 53 up- and 60 down-regulated genes in S1 were commonly detected in all 3 stages (Fig. 3). These core up- and down-regulated genes shared similar expression patterns among all three infected stages, and constituted the most basal expression response specific to the infection of *U. virens*. The conserved *cis*-acting elements may provide informative clues as to how to use plant responses to combat smut pathogen attack. Thus, we analyzed the upstream regions of these commonly regulated genes. One kb sequences in promoter regions of the 113 genes were obtained and analyzed using the PLACE *cis*-element database. The analysis revealed six and three enriched *cis-*acting elements for over 50% of the up- and down-regulated genes (P<0.05), respectively (Table 3). For the six conserved motifs in the promoter regions shared among the up-regulated genes, TTGAC and TGACT for W-box and TGTCA for Myb were both typically observed stress related motifs. In addition, the YTCANTYY motif acts as a basic transcription initiation element in some TATA-less promoters contributing to gene regulation. In some cases, it plays a role in the plant antioxidant system as a conserved *cis*-element of genes like monodehydroascorbate reductase, which is a crucial enzyme in the ascorbate-glutathione cycle [29]. The motif TAAAG is a typical binding site for Dof proteins, which are members of a major family of plant transcription factors involved in seed germination, phytohormones and defense responses [30]. Thus, these conserved *cis*-elements reflect a variety of roles in regulating the defense of host rice plants against invasion by the smut pathogen.

**Table 3:** *cis*-elements identified in the promoter region of the specially regulated genes

Regulated gene set	ID number of Motif	Ratio	*p* value	Conserved nucleotide	Annotation
Up-regulation	S000203	82%	0.0061	TTATTT	TATA like box for glutamine synthetase
	S000387	78%	0.0473	TAAAG	Dof gene binding
	S000390	76%	0.0043	TTGAC	WRKY DNA binding proteins
	S000395	72%	0.0148	YTCANTYY	Initiator/light-responsive transcription
	S000442	64%	0.0499	TGACT	SUSIBA2 bind to W-box element
	S000180	62%	0.0377	TGTCA	Myb DNA binding
Down-regulation	S000144	96.7%	0.0407	CANNTG	ABA-responsive
	S000501	65%	0.0003	VCGCGB	Calmodulin
	S000264	63.3%	0.0343	CATGCA	ABA-responsive

Note: Y stands for [CT], ie C or T; V stands for [GAC], ie G, A or C; B stands for [GTC], ie G, T or C; N stands for [ATGC], ie A, T, G or C. P value represents the significance between commonly detected up- and down-regulated DEGs.

For the down-regulated genes, two (CANNTG and CATGCA) out of three motifs were identified as cis-elements responding to abscisic acid (ABA). In rice, ABA has been demonstrated to respond to *M. grisea* infection and is antagonistic to both the salicylic acid (SA) and ethylene (ET) signaling pathways [11],[31]. We further explored the role of ABA in the regulation of gene expression during *U. virens* infections. ABA content in the florets was determined at different stages of *U. virens* invasion. At stage S3, ABA content was significantly reduced compared to that of the control (Fig. S4 in File S1), suggesting that ABA may play a critical role in mediating *U. virens* invasion in rice. The CGCG box is regulated by calmodulin and has been confirmed to be involved in the transcriptional regulation of plant defenses in response to pathogens [32]. In this study, the expression levels of 6 calmodulin related genes were specifically suppressed during *U. virens* infection at stages S1 to S3 (Fig. S5 in File S1), revealing an important role for calmodulins in pathogenic invasion by *U. virens*.

### Highly regulated unique genes analysis and pollen development play a key role in the rice-*U. virens* interaction

**Figure 5:**
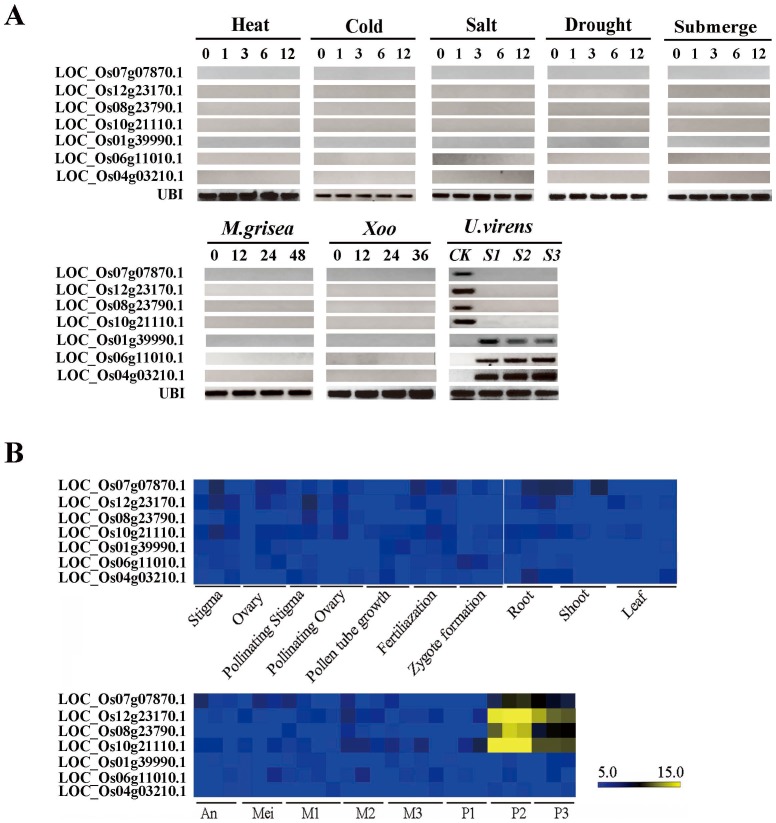
Expression pattern of seven uniquely responded genes under various stimuli. (**A**) RT-PCR analysis under different biotic and abiotic stress factors. 0, 1, 3, 6, 12, 24, 36 and 48 represent the hours of treatment. CK means of the spikelets. *UBI* was used as internal control. (**B**) Tissue specific expression based on public Microarray database. An, anther; Mei, Meiotic stage; M1, pollen mother cells at meiotic leptotene stage; M2, pollen mother cells at meiotic zygotene-pachytene stage; M3, pollen mother cells at meiotic diplotene-tetrad stage; P1, uni-nucleate pollen; P2, bi-cellular pollen; P3, tri-cellular mature pollen. The scale is transcript abundance log_2_ normalised values.

A plant's initial response to infection in plants is dependent on the recognition of pathogen-associated molecular patterns by a few unique genes of the host plant [33]. To find these pathogen receptors we analyzed 113 core unique genes (53 up and 60 down) with over a 5-fold change in expression over the course of the three stages of infection in Fig. 3. We also compared these highly modified genes to control plant expression databases measured in response to biotic and abiotic stressors. In total, 19 up-regulated and 10 repressed genes fit these criteria (Table S3 in File S1). These were further investigated under different treatments using the RT-PCR method. Finally, seven genes showed unique responses to the infection of *U. virens* (Fig. 5).

Of these, three genes that are not normally expressed in any rice tissue under normal or bio/abiotic stresses were uniquely activated after infection by *U. virens* (Fig. 5). LOC_Os01g39990.1 encodes an NBS-LRR-like protein, with homology to an *Arabidopsis* NB-ARC domain-containing disease resistance protein (AT3G14470) that has been confirmed to be involved in apoptosis. LOC_Os04g03210.1 encodes an LRR receptor kinase of unknown function, which is supposed to play a role in such processes as hormone reception and pathogen responses via protein-protein interactions of their conserved leucine-rich motifs and intracellular protein kinase domains with Ser/Thr specificity [34]. LOC_Os06g11010.1 encodes an aspartyl protease that is implicated in protein processing and/or degradation in plant senescence, stress responses, and programmed cell death and reproduction [35]. Their gene structure and unique expression patterns imply their specific roles in signaling the basic defense responses of rice to infection by the smut pathogen.

In contrast, LOC_Os07g07870.1, LOC_Os08g23790.1, LOC_Os12g23170.1 and LOC_Os10g21110.1, which encode the protease inhibitor, exopolygalacturonase, beta-glucosidase and glycosylhydrolase, respectively, were completely repressed (Fig. 5A). They are expressed only during late pollen development according to tissue expression patterns available in open microarray data, and had nothing to do with responses to heat, cold, salt, drought, submergence, *M. grisea* and *Xoo* infection (Fig. 5B). Their temporospatial expression patterns and their unique response to smut pathogen implicate their roles in both pollen development and in the establishment of the host-pathogen relationship.

The false smut pathogen disease phenotype occurs only in the spikelet during the late pollen development stage (Fig. 1). This means that other pollen or anther development related genes could be involved in the unique rice-*U.virens* interaction, and they may play critical roles in the signaling of downstream defense events [36],[37].

### Host carbohydrate dynamics in response to *U.virens* infection

**Figure 6:**
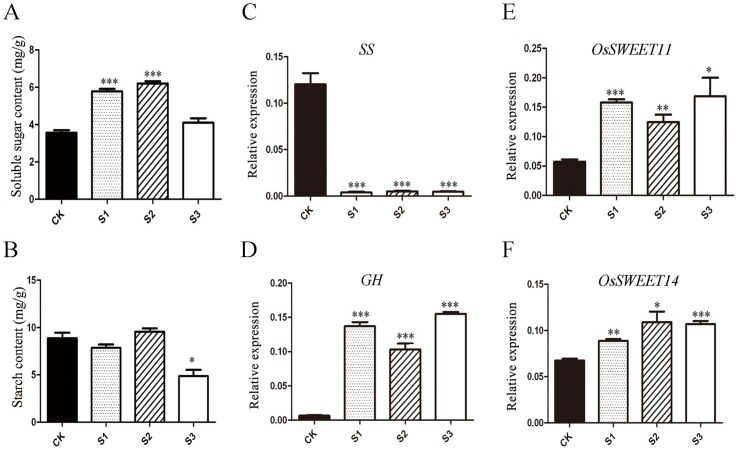
Content of soluble sugar and starch and expression of the related genes. **A–B**, Content of soluble sugar and starch in spiklets. **C–D**, qRT-PCR for some key genes in starch metabolism and carbohydrate transformation. **E–F**, qRT-PCR for two sugar transporter. *UBI* was used as the internal control. *, **, *** represented 0.05, 0.01 and 0.001 significant difference to the control, respectively.

Based on lifestyles, plant pathogens are often categorized into biotrophs and necrotrophs [38]. Generally, necrotrophs destroy infected sites and obtain nutrients from the dead cells, whereas biotrophs get organic carbon and other nutrition from hosts by generating penetration pegs, which can breach the host epidermal cells and form specialized structures like haustoria. One previous study showed that *U.virens* is a typical biotroph [7]. To further illuminate nutrition flow between host plants and *U.virens*, we analyzed the change of starch and soluble sugar metabolism between mock and infected spikelets. Results showed that the content of soluble sugars increased significantly in stages S1 and S2, but starch content was significantly reduced in S3 (Fig. 6A–B). In agreement with these results, a decrease in the expression level of starch synthase (*SS*) and an increase in the expression of the starch-degrading enzyme glycosyl hydrolase (*GH*) were observed in the infected spikelets (Fig. 6C–D). SWEETs are a member of family involved in carbohydrate flow between host plant and pathogen [39]. In the current study, two members of this family, *OsSWEET11* and *OsSWEET14*, were highly up-regulated during *U.virens* infection (Fig. 6E–F).

## Discussion

**Figure 7:**
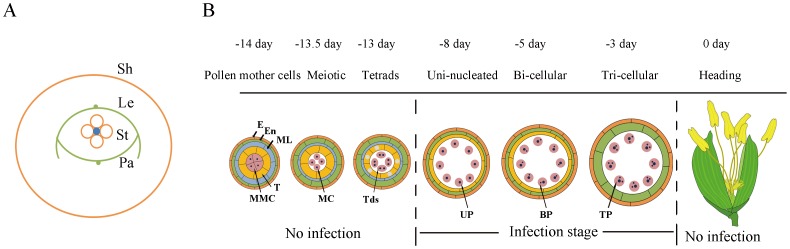
Diagrams of the rice pollen development and infection of *U. virens*. (**A**) Structural illustration of rice sheath at booting stage. Sh, Sheath; Le, lemma; Pa, palea; St, stamen. (**B**) Diagram of rice pollen development. E, epidermis; En, endothecium; ML, middle layer; T, tapetum; MMC, microspore mother cell; MC, meiotic cell; Tds, tetrads; UP, unicleate pollen; BP, bicellular pollen; TP, tricellular pollen.

Rice false smut is a fungal disease that results from the infection of plant reproductive tissues. Although it has been suggested that rice smut pathogen can infect rice roots and coleoptiles at the seedling stage, leading to asymptomatic colonization of the entire plant, no visual smut ball is formed from such an infection [7],[40],[41]. Upon investigation of the structure of rice reproductive organs, it is observed that microspores are tightly enclosed by three layers of physically-separated organs: flag leaf sheath, grain glume, and anthers, which protect and nourish the pollen grains during growth and development (Fig. 7A). Each of these three organs was found to be involved in different stages of infection by the smut pathogen: spore germination, hyphae colonization, and hyphae spread and sporulation respectively [42]. Conidia of the rice false smut pathogen enter into rice leaf sheaths along with rain drops or other vectors during the booting stage, after which germination occurs on the surface of grain glumes. At this point, hyphae invade the spikelets through the small gap between the lemma and palea at the apices to colonize and spread [42], finally intruding into the anther chambers by penetrating through the anther wall at the weak connectivum for sporulation (Fig. S1C–D in File S1). However, previous studies do not provide detailed information about which tissues the hyphae invade first. As booting panicles increase in complexity, it is hard to exactly judge which spikelets were infected after inoculation. Thus, the general “time post inoculation” parameter thatis widely used in other pathogenic systems did not fit for rice false smut. In the present study, we found that the infection of *U. virens* was a stepwise invasion process, which can be divided into three distinctive stages based on morphology of the infected floral organs (S1–S3). We further observed hyphae on the stigma, anther surface and anther chambers, but conidia only around microspores (Fig. 1), suggesting that only anther chambers can provide the smut pathogen with the most appropriate nutrients and space for the reproduction of conidia and formation of the smut ball (Fig. 1H, K).

It is generally accepted that infection with rice false smut occurs only at the booting stage, about 6–9 days before heading [7]. Inoculation with the pathogen at other stages is not able to cause disease or symptoms. It should be noted that this stage just overlaps with the development of pollen from the uni-nucleate to tri-cellular stages as shown in Fig. 7B [15]. At this stage, anther chambers are greatly enlarged and starches start to accumulate in pollen. We propose that the anther chambers specifically at this stage provide an appropriate space, and maturing pollen provides enough sugars and other nutrients to allow for smut pathogen growth and reproduction. To test this hypothesis, we analyzed carbohydrate metabolism dynamics between mock and disease spikelets. Results showed that with *U. virens* infection, the starch content showed no obvious change in S1∼S2 but greatly dropped in S3. However, soluble sugar content notably decreased in S3, compared with S1 and S2. It is notable that compare with starch, soluble sugars could be easily uptaken by pathogen. In the current study, we detected two SWEET members, *OsSWEET11* and *OsSWEET14*, were highly induced. These proteins are thought to be involved in the movement of sugar from the host cell to the pathogen in *Xoo* infection [39]. Thus, we believe that the combined evidence of alterations in the expression of sugar-related enzymes and starches in anthers, the production of sugars during pollen development, and the mass production of conidia in anther chambers during smut pathogen infection can partly explain why infection by the smut pathogen is limited to such a short period during the development of rice plants.

**Figure 8:**
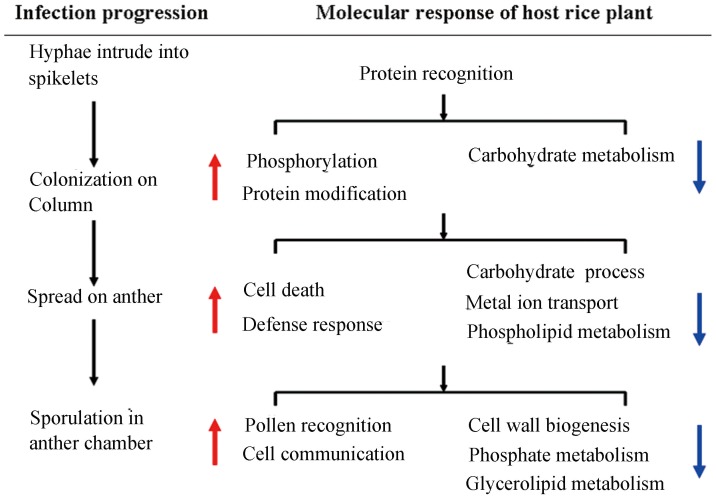
Biological process of rice plant response to the infection of *U. virens*. Red arrow represents up-regulation BP terms; Blue arrow represents down-regulation BP terms.

Thousands of genes are regulated as part of an intricate and complex network in plants' responses to various biotic and abiotic stimuli [43],[44]. According to the evolutionary theory of plant-pathogen interactions, apart from the general defense systems related to innate immunity, each plant species has developed its own special genes to mediate the compatible/incompatible plant-microbe interactions of certain pathogens [45]. However, the multitude of information obtained from these studies makes it difficult to identify which genes and signaling pathways are of key importance in host-pathogen interactions, thus investigation of the molecular responses to various stressors has focused primarily on the common transcriptional patterns between them [46]. In the present study, spikelets were sampled from different inoculated panicles at three infection stages and further analyzed by Illumina HiSeq 2000 between two years. Hundreds of genes that were induced in rice plants following rice false smut infection were carefully evaluated. General bio-/abiotic stress-related genes available in open databases were then eliminated in order to identify specific genes differentially expressed in response to rice false smut infection. Genes thought to be involved in the plant's response to this pathogen were induced in increasing numbers in a series-specific manner as the disease progressed from S1 to S3 (Fig. 8).

In the infection stage S1, the pathogen *U. virens* colonized on the styles of the pistil. At this stage, biological processes related to phosphorylation and protein modification were greatly activated in the infected spikelet, indicating their involvement in the defense response to the invasion of the rice smut pathogen. These proteins are involved in protein modification, protein degradation and receptor phosphorylation etc. (Fig. 4A). As a widespread post-translational protein modification, phosphorylation participates in plant defense signal transduction in at least two known aspects [47]. First, some receptor protein kinases can activate corresponding substrates to facilitate downstream signal transduction. One example of this is shown by MPK3 and MPK6, which phosphorylate WRKY33 to initiate phytoalexin biosynthesis in *Arabidopsis*. Secondly, pathogen effectors can conceal conserved phosphorylation sites in the activation receptor, reducing their kinase activity, and consequently inhibiting downstream immune signaling [48]. Of these, LOC_Os01g02310.1, a receptor-like kinase, has been shown to interact with *OsEBP-89*, an EREBP transcription factor downstream of the JA/ETH pathway, and can be widely induced by various stresses [49],[50] (Fig. 4A). LRR XI is a subgroup of the LRR super family that includes *Xa21*, a resistant gene for *Xoo* [51]. WAKs is a subfamily of the cell wall associated kinase family, some members of which have been shown to signal upstream of general recognition defense signaling pathways [52]. During stages S2 and S3, the up-regulated genes appear to be involved in biological processes of “Cell death” (Fig. 8), a common innate immune response of plant cells to pathogen invasion. This response may be the result of downstream signaling resulting from the activation of “Proteolysis” processes in stage S1, because plant proteases have been suggested as being involved in multiple pathogen defenses by activating hypersensitive responses and programmed cell death [53]. This hypothesis corresponds to our observations that *U. virens* hyphae pierced into anther chambers through anther connectivum, and brought about pollen collapse as shown in Fig. S1 in File S1.

As the disease progresses, biological processes crucial for plant development such as “Carbohydrate metabolic process”, “Ion transport” and “Cellular lipid metabolic process” etc. were widely repressed at stage S2 (Fig. 8). This suggests that infection by *U. virens* pathogen inhibits the normal growth and development of plant floral organs. Following pathogen entry into the anther chambers at stage S3, processes such as “Cell wall organization”, “Glycerolipid metabolic process”, “Protein modification” and “Protein amino acid phosphorylation” were extensively down-regulated (Fig. 8). LOC_Os05g38770.1 encodes protein kinase APK1B (Fig. S5 in File S1). In *Arabidopsis*, APK1B is involved in stamen development, and its repression can prevent pollen tube germination causing self incompatibility [54]. This data is in accordance with the observation that pollen surfaces were fully wrapped by masses of hyphae and conidia at this stage (Fig. 1H), suggesting that the pathogen manipulates host development signaling by prohibiting protein phosphorylation, hence allowing further infection of the plant with *U. virens* to occur.

## Supporting Information

File S1 **Figure S1.** Scanning electronic microscope observation rice false smut. **Figure S2.** Sequencing saturation analysis about eight DGE libraries. **Figure S3.** Comparison analysis and verification of the regulated DGE genes. **Figure S4.** Quantitation of ABA content. **Figure S5.** Heat map of the stage-specific genes involved in BP terms of “protein modification” in down regulation part. **Table S1.** RNA-Seq reads in eight DGE libraries alignment to reference datasetbase. **Table S2.** Primers used in this paper. **Table S3.** Highly regulated genes with expression level over 5-fold in stage S1∼S3.
**Figure S1 Scanning electronic microscope observation rice false smut.** **A.** Observation of the infected floral organs at stage S1. **B-D.** Observation of the infected floral organs at stage S3. Hy indicates hyphae; Sl showed sheet-like structures. Bar=100 mm in Fig. A∼B; Bar=10 mm in Fig. C∼D.
**Figure S2. Sequencing saturation analysis about eight DGE libraries.** **A.** CK-S3 libraries for 2010 sample; **B.** CK-S3 libraries for 2011 sample. The y-axis indicates perc
**Figure S3. Comparison analysis and verification of the regulated DGE genes.** **A.** The number of up- and down-regulation genes differentially expressed among three infected stages in 2010 and 2011. **B.** Several genes stand for major functional categories in two years were randomly selected for analysis. Changes in gene expression represented as log2 derived from qPCR and DGE data. Error bars for qRT-PCR show the standard deviation of three replicates.
**Figure S4. Quantitation of ABA content.** The data were shown as mean±SD (n= 3), *, **, *** represented 0.05, 0.01 and 0.001 significant difference to the control, respectively.
**Figure S5. Heat map of the stage-specific genes involved in BP terms of “protein modification” in down regulation part.** Colours bar represent expression levels of each gene which are either up-regulated (red) or down-regulated (blue).
**Table S1. RNA-Seq reads in eight DGE libraries alignment to reference datasetbase.**
**Table S2. Primers used in this paper.**
**Table S3. Highly regulated genes with expression level over 5-fold in stage S1∼S3.**
(ZIP)
