# Supplementary figures and images for: Cytological and Transcriptional Dynamics Analysis of Host Plant Revealed Stage-Specific Biological Processes Related to Compatible Rice-Ustilaginoidea virens Interaction
Source: PLoS One. 2014 Mar 19;9(3):e91391. doi: 10.1371/journal.pone.0091391 (PMC3960121; doi:10.1371/journal.pone.0091391)

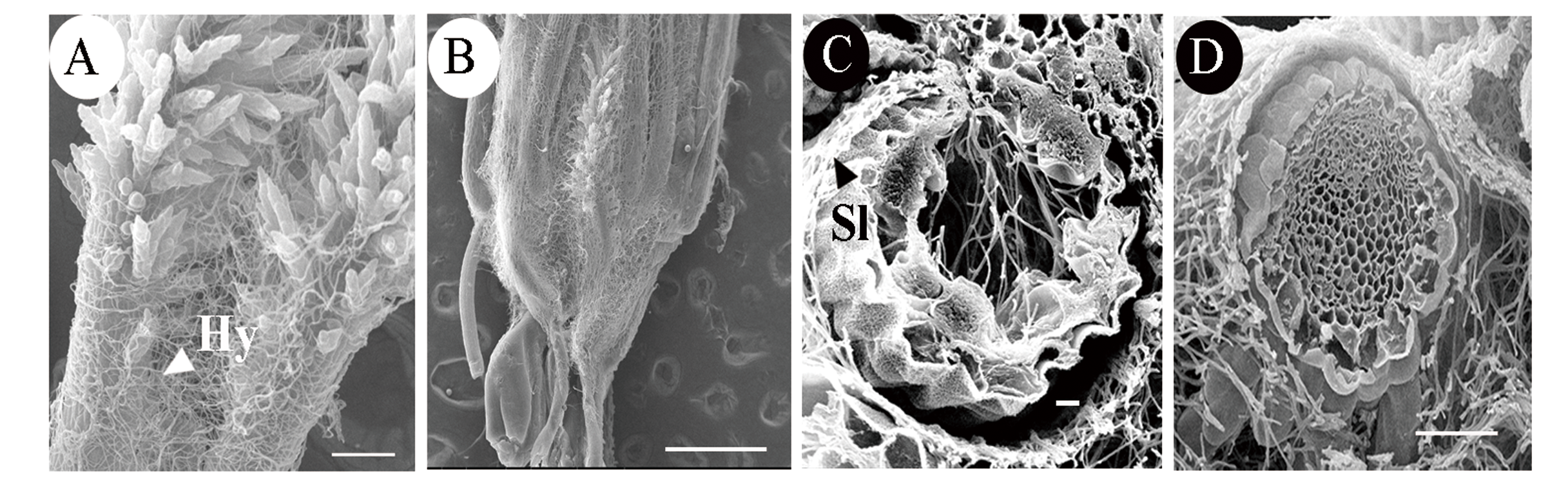

Supplement: File S1 — Figure S1. Scanning electronic microscope observation rice false smut. Figure S2. Sequencing saturation analysis about eight DGE libraries. Figure S3. Comparison analysis and verification of the regulated DGE genes. Figure S4. Quantitation of ABA content. Figure S5. Heat map of the stage-specific genes involved in BP terms of “protein modification” in down regulation part. Table S1. RNA-Seq reads in eight DGE libraries alignment to reference datasetbase. Table S2. Primers used in this paper. Table S3. Highly regulated genes with expression level over 5-fold in stage S1∼S3. Figure S1 Scanning electronic microscope observation rice false smut. A. Observation of the infected floral organs at stage S1. B-D. Observation of the infected floral organs at stage S3. Hy indicates hyphae; Sl showed sheet-like structures. Bar=100 mm in Fig. A∼B; Bar=10 mm in Fig. C∼D. Figure S2. Sequencing saturation analysis about eight DGE libraries. A. CK-S3 libraries for 2010 sample; B. CK-S3 libraries for 2011 sample. The y-axis indicates perc Figure S3. Comparison analysis and verification of the regulated DGE genes. A. The number of up- and down-regulation genes differentially expressed among three infected stages in 2010 and 2011. B. Several genes stand for major functional categories in two years were randomly selected for analysis. Changes in gene expression represented as log2 derived from qPCR and DGE data. Error bars for qRT-PCR show the standard deviation of three replicates. Figure S4. Quantitation of ABA content. The data were shown as mean±SD (n= 3), *, **, *** represented 0.05, 0.01 and 0.001 significant difference to the control, respectively. Figure S5. Heat map of the stage-specific genes involved in BP terms of “protein modification” in down regulation part. Colours bar represent expression levels of each gene which are either up-regulated (red) or down-regulated (blue). Table S1. RNA-Seq reads in eight DGE libraries alignment to reference datasetbase. Table S2. Primers us [file pone.0091391.s001.zip › Figure S1.tif]

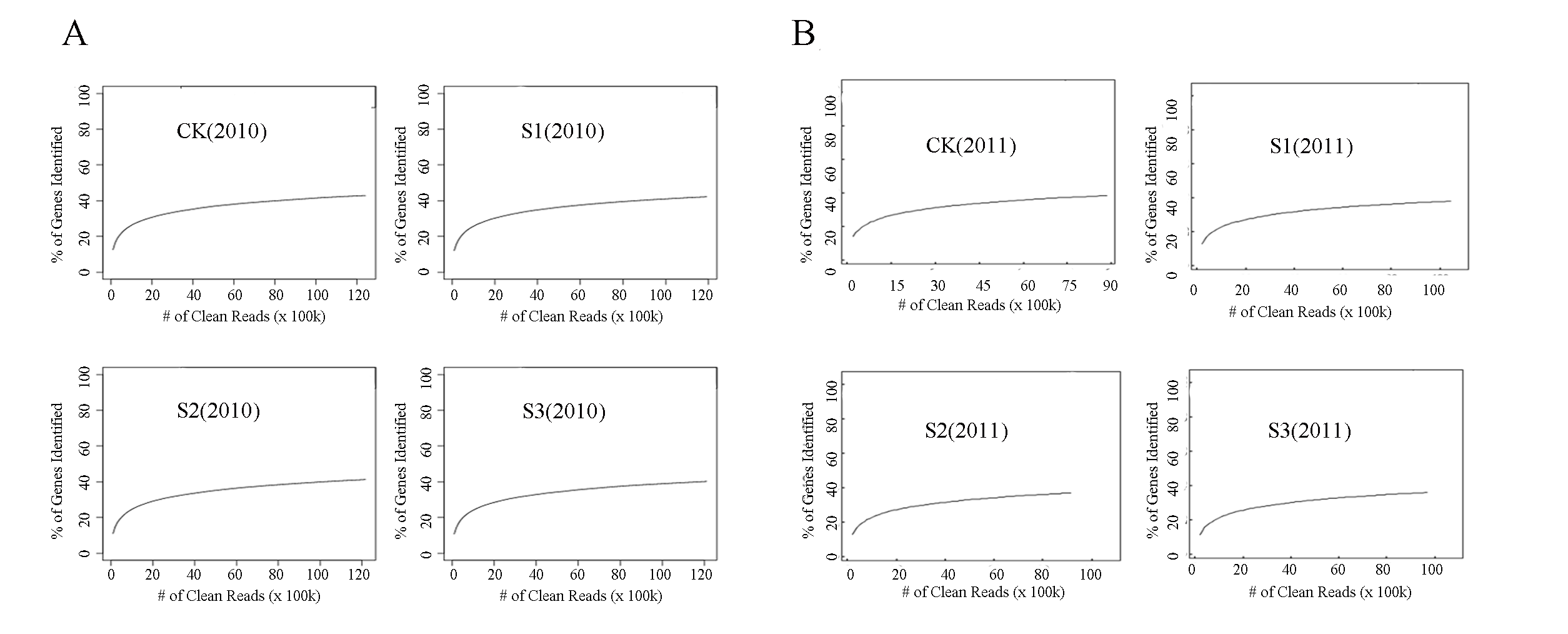

Supplement: File S1 — Figure S1. Scanning electronic microscope observation rice false smut. Figure S2. Sequencing saturation analysis about eight DGE libraries. Figure S3. Comparison analysis and verification of the regulated DGE genes. Figure S4. Quantitation of ABA content. Figure S5. Heat map of the stage-specific genes involved in BP terms of “protein modification” in down regulation part. Table S1. RNA-Seq reads in eight DGE libraries alignment to reference datasetbase. Table S2. Primers used in this paper. Table S3. Highly regulated genes with expression level over 5-fold in stage S1∼S3. Figure S1 Scanning electronic microscope observation rice false smut. A. Observation of the infected floral organs at stage S1. B-D. Observation of the infected floral organs at stage S3. Hy indicates hyphae; Sl showed sheet-like structures. Bar=100 mm in Fig. A∼B; Bar=10 mm in Fig. C∼D. Figure S2. Sequencing saturation analysis about eight DGE libraries. A. CK-S3 libraries for 2010 sample; B. CK-S3 libraries for 2011 sample. The y-axis indicates perc Figure S3. Comparison analysis and verification of the regulated DGE genes. A. The number of up- and down-regulation genes differentially expressed among three infected stages in 2010 and 2011. B. Several genes stand for major functional categories in two years were randomly selected for analysis. Changes in gene expression represented as log2 derived from qPCR and DGE data. Error bars for qRT-PCR show the standard deviation of three replicates. Figure S4. Quantitation of ABA content. The data were shown as mean±SD (n= 3), *, **, *** represented 0.05, 0.01 and 0.001 significant difference to the control, respectively. Figure S5. Heat map of the stage-specific genes involved in BP terms of “protein modification” in down regulation part. Colours bar represent expression levels of each gene which are either up-regulated (red) or down-regulated (blue). Table S1. RNA-Seq reads in eight DGE libraries alignment to reference datasetbase. Table S2. Primers us [file pone.0091391.s001.zip › Figure S2.tif]

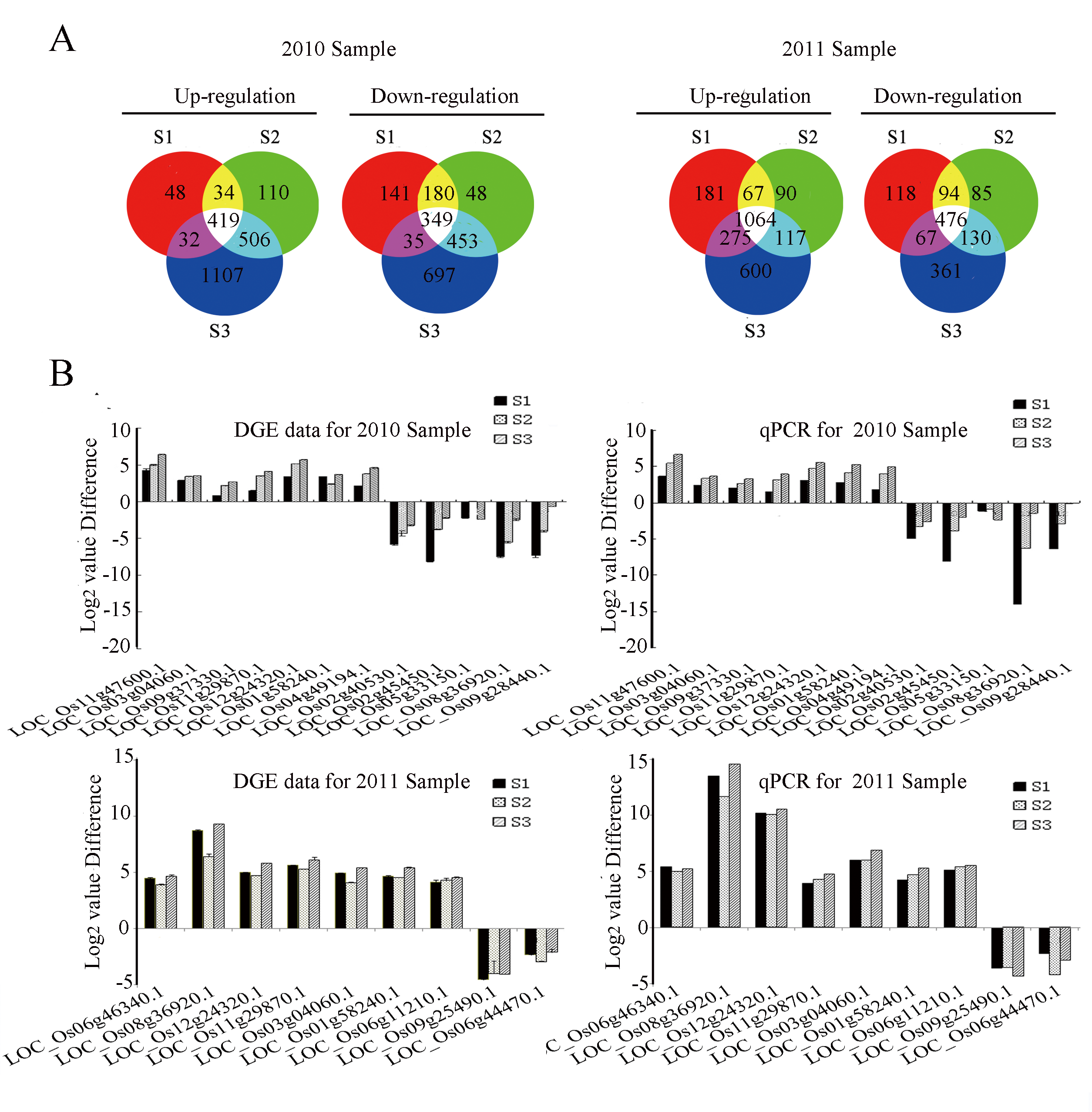

Supplement: File S1 — Figure S1. Scanning electronic microscope observation rice false smut. Figure S2. Sequencing saturation analysis about eight DGE libraries. Figure S3. Comparison analysis and verification of the regulated DGE genes. Figure S4. Quantitation of ABA content. Figure S5. Heat map of the stage-specific genes involved in BP terms of “protein modification” in down regulation part. Table S1. RNA-Seq reads in eight DGE libraries alignment to reference datasetbase. Table S2. Primers used in this paper. Table S3. Highly regulated genes with expression level over 5-fold in stage S1∼S3. Figure S1 Scanning electronic microscope observation rice false smut. A. Observation of the infected floral organs at stage S1. B-D. Observation of the infected floral organs at stage S3. Hy indicates hyphae; Sl showed sheet-like structures. Bar=100 mm in Fig. A∼B; Bar=10 mm in Fig. C∼D. Figure S2. Sequencing saturation analysis about eight DGE libraries. A. CK-S3 libraries for 2010 sample; B. CK-S3 libraries for 2011 sample. The y-axis indicates perc Figure S3. Comparison analysis and verification of the regulated DGE genes. A. The number of up- and down-regulation genes differentially expressed among three infected stages in 2010 and 2011. B. Several genes stand for major functional categories in two years were randomly selected for analysis. Changes in gene expression represented as log2 derived from qPCR and DGE data. Error bars for qRT-PCR show the standard deviation of three replicates. Figure S4. Quantitation of ABA content. The data were shown as mean±SD (n= 3), *, **, *** represented 0.05, 0.01 and 0.001 significant difference to the control, respectively. Figure S5. Heat map of the stage-specific genes involved in BP terms of “protein modification” in down regulation part. Colours bar represent expression levels of each gene which are either up-regulated (red) or down-regulated (blue). Table S1. RNA-Seq reads in eight DGE libraries alignment to reference datasetbase. Table S2. Primers us [file pone.0091391.s001.zip › Figure S3.tif]

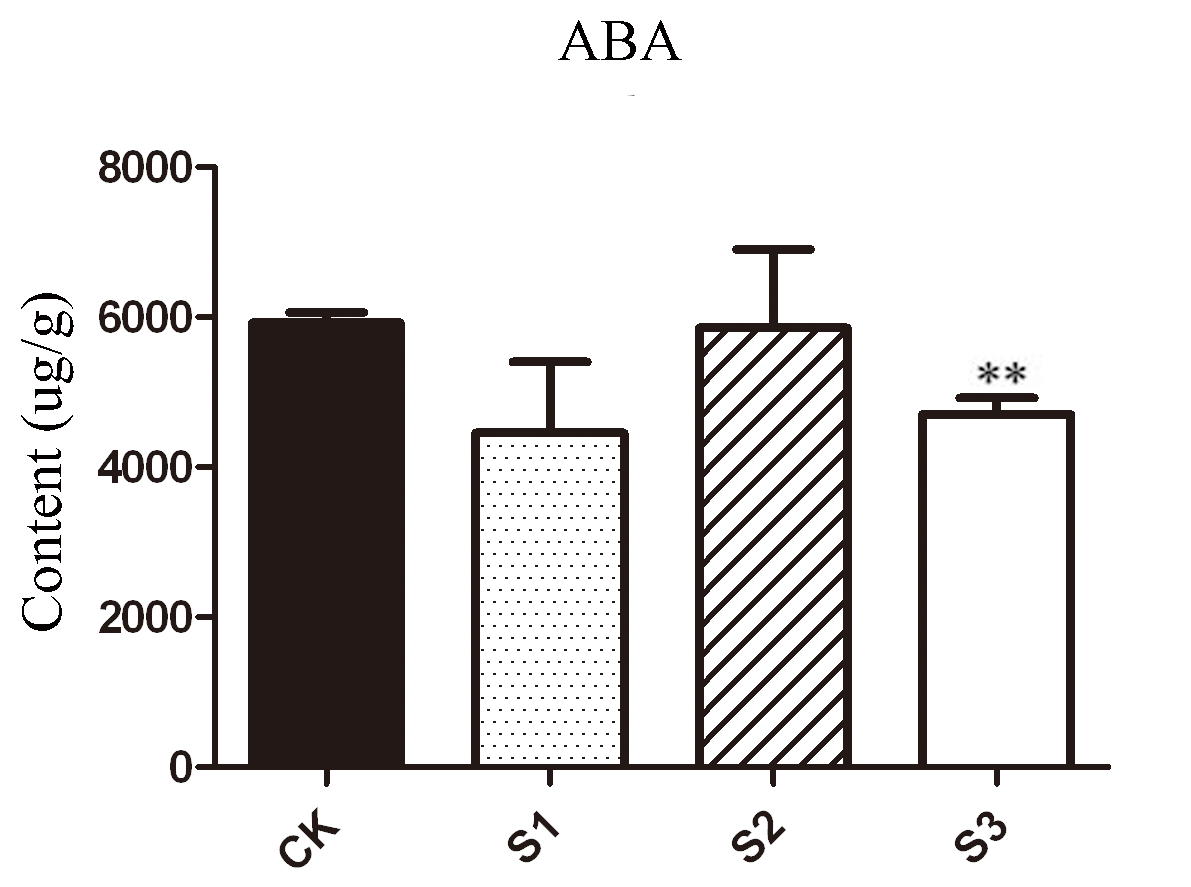

Supplement: File S1 — Figure S1. Scanning electronic microscope observation rice false smut. Figure S2. Sequencing saturation analysis about eight DGE libraries. Figure S3. Comparison analysis and verification of the regulated DGE genes. Figure S4. Quantitation of ABA content. Figure S5. Heat map of the stage-specific genes involved in BP terms of “protein modification” in down regulation part. Table S1. RNA-Seq reads in eight DGE libraries alignment to reference datasetbase. Table S2. Primers used in this paper. Table S3. Highly regulated genes with expression level over 5-fold in stage S1∼S3. Figure S1 Scanning electronic microscope observation rice false smut. A. Observation of the infected floral organs at stage S1. B-D. Observation of the infected floral organs at stage S3. Hy indicates hyphae; Sl showed sheet-like structures. Bar=100 mm in Fig. A∼B; Bar=10 mm in Fig. C∼D. Figure S2. Sequencing saturation analysis about eight DGE libraries. A. CK-S3 libraries for 2010 sample; B. CK-S3 libraries for 2011 sample. The y-axis indicates perc Figure S3. Comparison analysis and verification of the regulated DGE genes. A. The number of up- and down-regulation genes differentially expressed among three infected stages in 2010 and 2011. B. Several genes stand for major functional categories in two years were randomly selected for analysis. Changes in gene expression represented as log2 derived from qPCR and DGE data. Error bars for qRT-PCR show the standard deviation of three replicates. Figure S4. Quantitation of ABA content. The data were shown as mean±SD (n= 3), *, **, *** represented 0.05, 0.01 and 0.001 significant difference to the control, respectively. Figure S5. Heat map of the stage-specific genes involved in BP terms of “protein modification” in down regulation part. Colours bar represent expression levels of each gene which are either up-regulated (red) or down-regulated (blue). Table S1. RNA-Seq reads in eight DGE libraries alignment to reference datasetbase. Table S2. Primers us [file pone.0091391.s001.zip › Figure S4.tif]

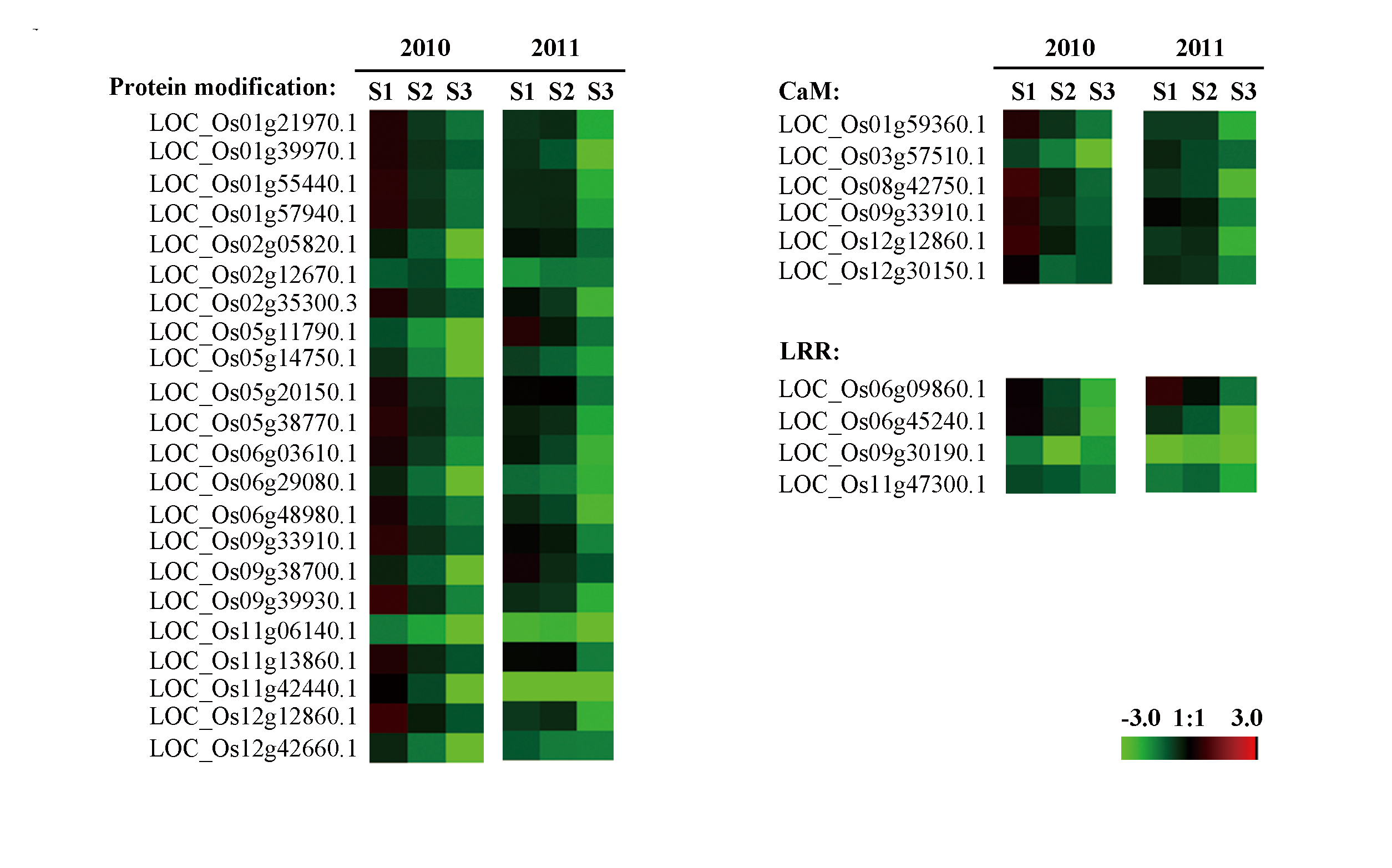

Supplement: File S1 — Figure S1. Scanning electronic microscope observation rice false smut. Figure S2. Sequencing saturation analysis about eight DGE libraries. Figure S3. Comparison analysis and verification of the regulated DGE genes. Figure S4. Quantitation of ABA content. Figure S5. Heat map of the stage-specific genes involved in BP terms of “protein modification” in down regulation part. Table S1. RNA-Seq reads in eight DGE libraries alignment to reference datasetbase. Table S2. Primers used in this paper. Table S3. Highly regulated genes with expression level over 5-fold in stage S1∼S3. Figure S1 Scanning electronic microscope observation rice false smut. A. Observation of the infected floral organs at stage S1. B-D. Observation of the infected floral organs at stage S3. Hy indicates hyphae; Sl showed sheet-like structures. Bar=100 mm in Fig. A∼B; Bar=10 mm in Fig. C∼D. Figure S2. Sequencing saturation analysis about eight DGE libraries. A. CK-S3 libraries for 2010 sample; B. CK-S3 libraries for 2011 sample. The y-axis indicates perc Figure S3. Comparison analysis and verification of the regulated DGE genes. A. The number of up- and down-regulation genes differentially expressed among three infected stages in 2010 and 2011. B. Several genes stand for major functional categories in two years were randomly selected for analysis. Changes in gene expression represented as log2 derived from qPCR and DGE data. Error bars for qRT-PCR show the standard deviation of three replicates. Figure S4. Quantitation of ABA content. The data were shown as mean±SD (n= 3), *, **, *** represented 0.05, 0.01 and 0.001 significant difference to the control, respectively. Figure S5. Heat map of the stage-specific genes involved in BP terms of “protein modification” in down regulation part. Colours bar represent expression levels of each gene which are either up-regulated (red) or down-regulated (blue). Table S1. RNA-Seq reads in eight DGE libraries alignment to reference datasetbase. Table S2. Primers us [file pone.0091391.s001.zip › Figure S5.tif]
