# Supplementary material for: Cytological and Transcriptional Dynamics Analysis of Host Plant Revealed Stage-Specific Biological Processes Related to Compatible Rice-Ustilaginoidea virens Interaction
Source: PLoS One. 2014 Mar 19;9(3):e91391. doi: 10.1371/journal.pone.0091391 (PMC3960121; doi:10.1371/journal.pone.0091391)
Supplement: File S1 — Figure S1. Scanning electronic microscope observation rice false smut. Figure S2. Sequencing saturation analysis about eight DGE libraries. Figure S3. Comparison analysis and verification of the regulated DGE genes. Figure S4. Quantitation of ABA content. Figure S5. Heat map of the stage-specific genes involved in BP terms of “protein modification” in down regulation part. Table S1. RNA-Seq reads in eight DGE libraries alignment to reference datasetbase. Table S2. Primers used in this paper. Table S3. Highly regulated genes with expression level over 5-fold in stage S1∼S3. Figure S1 Scanning electronic microscope observation rice false smut. A. Observation of the infected floral organs at stage S1. B-D. Observation of the infected floral organs at stage S3. Hy indicates hyphae; Sl showed sheet-like structures. Bar=100 mm in Fig. A∼B; Bar=10 mm in Fig. C∼D. Figure S2. Sequencing saturation analysis about eight DGE libraries. A. CK-S3 libraries for 2010 sample; B. CK-S3 libraries for 2011 sample. The y-axis indicates perc Figure S3. Comparison analysis and verification of the regulated DGE genes. A. The number of up- and down-regulation genes differentially expressed among three infected stages in 2010 and 2011. B. Several genes stand for major functional categories in two years were randomly selected for analysis. Changes in gene expression represented as log2 derived from qPCR and DGE data. Error bars for qRT-PCR show the standard deviation of three replicates. Figure S4. Quantitation of ABA content. The data were shown as mean±SD (n= 3), *, **, *** represented 0.05, 0.01 and 0.001 significant difference to the control, respectively. Figure S5. Heat map of the stage-specific genes involved in BP terms of “protein modification” in down regulation part. Colours bar represent expression levels of each gene which are either up-regulated (red) or down-regulated (blue). Table S1. RNA-Seq reads in eight DGE libraries alignment to reference datasetbase. Table S2. Primers us [file pone.0091391.s001.zip › Table S2.doc]

**Table S2	Primer**s used in this paper

| LOC_ID | Primer | Description | Optimum temperature |
| --- | --- | --- | --- |
| LOC_Os11g47600.1 | Forward:CGCCATTGCTGCTTCATCG  Reverse:CACTCACAAATTACATACGC | Glycosyl hydrolase | 55℃ |
| LOC_Os03g04060.1 | Forward:GTCGTCATAAGCGGATTC  Reverse:GTGAGATAAGCGACCATG | CHIT16 | 49℃ |
| LOC_Os09g37330.1 | Forward:CTGAAGCACCAATACACT  Reverse:CTTTAGTTGCTCCCGTTA | OsSAUR39 | 59℃ |
| LOC_Os11g29870.1 | Forward:TGCATGGATCGATGGATGTTA  Reverse:GAATCTTGCTTGCCACTGAATCT | OsWRKY72 | 59℃ |
| LOC_Os12g24320.1 | Forward:CGCGTGGTGTTTGGTTTC  Reverse:CATGCAGTTACTCTGTCCC | ATPase 3 | 57℃ |
| LOC_Os01g58240.1 | Forward:ATAACCTGGGCAAAACATG  Reverse:ATTGGCCGGTCATTAACTC | OsSub6 | 57℃ |
| LOC_Os04g49194.1 | Forward:ACTTGCGCTTCACTACCC  Reverse:AAAGCATTGCCCGACATA | Naringenin,2-oxoglutarate 3-dioxygenase | 59℃ |
| LOC_Os02g40530.1 | Forward:CTCCGCTACAGGATCAAGG  Reverse:GGATGCACAAGACCAAAAG | MYB domain containing protein | 63℃ |
| LOC_Os02g45450.1 | Forward:GTAATGTTATGTATCGCTGCTC  Reverse:CACAGTGCCCGACTTTCT | Dehydration-responsive element-binding protein | 55℃ |
| LOC_Os05g33150.1 | Forward:TACGCACTTCGCACGGT  Reverse:GGGCTTTCACAGATCCA | CHIT6 | 55℃ |
| LOC_Os08g36920.1 | Forward:TGGTTCCCACAGTCGTCA  Reverse:TAGTACACCAGAGCCAAT | AP2 domain containing protein | 55℃ |
| LOC_Os09g28440.1 | Forward:AACCGTTGGATTAGATTAC  Reverse:CTCTGCACAAACAATGAAGTA | AP2 domain containing protein | 55℃ |
| LOC_Os06g44470.1 | Forward:GTTGAGTCGCATGAGAAT  Reverse:CAATGGACTATTACCGATGAA | Pollen allergen | 56℃ |
| LOC_Os09g25490.1 | Forward:TGAAGAGGGAATGGACAGAT  Reverse:AGTAGGAGCAATGAGAACAC | CESA9 - cellulose synthase | 56℃ |
| LOC_Os06g11210.1 | Forward:CTCGGTTGCTGATGCTTAGATT  Reverse:GCACGCAGGCATATACTAACA | 12-oxophytodienoate reductase | 58℃ |
| LOC_Os08g42350.1 | Forward:TCTCGCTACTGATGAAG  Reverse:ACTAGCTGCTAATGACA | OsSWEET11 | 58℃ |
| LOC_Os11g31190.1 | Forward:GCCGTAAGAAGGAAGAAG  Reverse:TTTAGTCGTCGCACTCTC | OsSWEET14 | 56℃ |
| LOC_Os06g04200.3 | Forward:GCTCGCCAAGGAGAACG  Reverse:CAGGGTCCGATAAGATA | Starch synthase (SS) | 61℃ |
| LOC_Os06g46340.1 | Forward:GTCGTCGGGCAGGAGTTC  Reverse:TCAGCTCAATTTCGTCGGGTTT | Glycosyl hydrolase (GH) | 61℃ |
| LOC_Os04g03210.1 | Forward:TGAGGATAGCGAAAGAGG  Reverse:CAAGCCCAAAGTCACCAA | Receptor kinase | 56℃ |
| LOC_Os11g31530.1 | Forward:CTCTCCCTTGTCCTTGTC  Reverse:ATTTTCCACTCCCATTTTCC | BRASSINOSTEROID INSENSITIVE 1-associated receptor kinase 1 | 60℃ |
| LOC_Os05g43090.1 | Forward:GCAACAGCACAGATGAAG Reverse:CTTGAGGCACCAACGATT | GDSL-like lipase/acylhydrolase | 60℃ |
| LOC_Os06g11010.1 | Forward:TGGGTATCACCGGACATCA Reverse:ATCTCCAGCAGAGGCAAGG | Eukaryotic aspartyl protease domain containing protein | 56℃ |
| LOC_Os12g36840.1 | Forward:TGCCGAGTACGCCTAAAA Reverse:CACCCACATGAAATCAAACA | Pathogenesis-related Bet v I family protein | 57℃ |
| LOC_Os03g37290.1 | Forward:GCGATTAGGTGTGAAATAAG Reverse:AACAACTCGTACCATCTG | Cytochrome P450 | 60℃ |
| LOC_Os10g31780.1 | Forward:CGTGAAGAAGGTGGAGGAGA Reverse:CGAAGCCGCCATTGACCC | Short chain dehydrogenase/reductase family domain containing protein | 60℃ |
| LOC_Os02g41904.1 | Forward:GCTTCTGTTCTTGGCTTAC Reverse:GCTGAAATGTGGAACCTTAC | DEF7 - Defensin and Defensin-like DEFL family | 56℃ |
| LOC_Os12g23170.1 | Forward:GTTGGATTATTATCACTGTTG Reverse:GATGTCCTGAATCTATTAAACTA | Os12bglu38 | 56℃ |
| LOC_Os01g28450.1 | Forward:CAACTTCGTCGGACAGAG Reverse:CGCTTTATAGAATCCTCGGTAT | SCP-like extracellular protein | 56℃ |
| LOC_Os04g45510.1 | Forward:TCGTCCCAGCAGAAATAG Reverse:GAATACCACAATGGCAAA | Expressed protein | 56℃ |
| LOC_Os03g52180.1 | Forward: GAGTAGGCGTTGTGAATC  Reverse: TGTTCTTGCTTGTTGTTCT | 4-hydroxy-3-methylbut-2-enyl diphosphate reductase | 56℃ |
| LOC_Os04g57840.1 | Forward: GTTCATCGCCAAGCTCCCCTC  Reverse: TTGCATTGCTGCTCGGTTCCT | Transposon protein | 56℃ |
| LOC_Os06g11970.1 | Forward: AATTAGATGCTTTTGTACT  Reverse: AGATGATAAATAACTCGAT | OsMADS63 | 53℃ |
| LOC_Os02g25860.1 | Forward: AAATCCTGCTAATCCCACA  Reverse: GCTCCTAGACTCCTCCCTT | Glutelin | 56℃ |
| LOC_Os04g37460.1 | Forward: TTTGCTGCTGCTTCTTTTCTT  Reverse: ATCTCACTTCTGTTCTGCCTG | Glutamate decarboxylase | 56℃ |
| LOC_Os10g05790.1 | Forward: CTCCGATCTACCGTCCCCCG  Reverse: TTCCCAAATGCCGTAAACAC | POEI4 | 56℃ |
| LOC_Os07g07870.1 | Forward: GTGCCTCTGCATGGTCCTG  Reverse: TGGTTCATCCTCGTTTGTC | LTPL77 - Protease inhibitor | 56℃ |
| LOC_Os10g21110.1 | Forward: CATTAAGGTCTAGGAGAGTGTGA  Reverse: GCAGTGGCTAGGCGTATA | Glycosyl hydrolase family 10 protein | 56℃ |
| LOC_Os01g39990.1 | Forward: CTGAAGAGGAGGAGAGGAATATG  Reverse: TGCTGTCATCACTTGGTATCA | Expressed protein | 56℃ |
| LOC_Os03g13170.1 | Forward:CGCAAGAAGAAGTGTGGTCA Reverse:ACGATTGATTTAACCAGTCCATGA | UBI | 59℃ |
